# Special Report on the International Temperature Scale of 1990: Report on the 17th Session of the Consultative Committee on Thermometry

**DOI:** 10.6028/jres.095.007

**Published:** 1990

**Authors:** B. W. Mangum

**Affiliations:** National Institute of Standards and Technology, Gaithersburg, MD 20899

**Keywords:** CCT, Comité Consultatif de Thermométrie, Consultative Committee on Thermometry, International Temperature Scale of 1990, temperature, temperature scale, thermodynamic temperature, thermometry

## Abstract

This article summarizes the results of the 17th Session of the Consultative Committee on Thermometry of the International Committee of Weights and Measures (Comité Consultatif de Thermométrie of the Comité International des Poids et Mesures) that met in Sèvres, France, September 12–14, 1989. That session was devoted exclusively to the completion of the International Temperature Scale of 1990, described herein, and to the implications of its adoption.

## 1. Introduction

The Consultative Committee on Thermometry (Comité Consultatif de Thermométrie, CCT) is one of eight Consultative Committees (Comités Consultatifs) of the International Committee of Weights and Measures (Comité International des Poids et Mesures, CIPM). The CIPM is a committee of the General Conference of Weights and Measures (Conférence Générale des Poids et Mesures, CGPM). The eight Consultative Committees (Comités Consultatifs) of the CIPM are:

1. The Comité Consultatif d’Électricité (CCE), established in 1927,
2. The Comité Consultatif de Photométrie et Radiométrie (CCPR), assigned this name in 1971; the previous name was the Comité Consultatif de Photométrie, established in 1933,
3. The Comité Consultatif de Thermométrie (CCT), established in 1937,
4. The Comité Consultatif pour la Définition du Métre (CCDM), established in 1952,
5. The Comité Consultatif pour la Définition de la Seconde (CCDS), established in 1956,
6. The Comité Consultatif pour les Étalons de Mesure des Rayonnements Ionisants (CCEMRI), established in 1958,
7. The Comité Consultatif des Unités (CCU), established in 1964, and
8. The Comité Consultatif pour la Masse et les grandeurs apparentées (CCM), established in 1980.

The CCT is composed presently of members from the following laboratories:

1. Amt für Standardisierung, Messwesen und Warenprüfung [ASMW], Berlin, DDR,
2. Bureau National de Métrologie, Paris, France: Institut National de Métrologie, [INM] du Conservatoire National des Arts et Métiers,
3. Ceskoslovensky Metrologicky Ustav [CSMU], Bratislava, Czechoslovakia,
4. National Research Council [NRC], Ottawa, Canada,
5. CSIRO, Division of Applied Physics [CSIRO], Lindfield, Australia,
6. D. I. Mendeleyev Institute for Metrology [VNIIM], Leningrad, USSR; Physico-Technical and Radio-Technical Measurements Institute (PRMI), Moscow, USSR,
7. National Institute of Metrology [NIM], Beijing, PRC,
8. Istituto di Metrologia G. Colonnetti [IMGC], Turin, Italy
9. Kamerlingh Onnes Laboratorium [KOL], Leiden, The Netherlands,
10. Korea Standards Research Institute [KSRI], Seoul, Korea,
11. National Institute of Standards and Technology [NIST], Gaithersburg, MD, USA
12. National Physical Laboratory [NPL], Teddington, UK,
13. National Research Laboratory of Metrology [NRLM], Ibaraki, Japan,
14. Physikalisch-Technische Bundesanstalt [PTB], Braunschweig, FRG,
15. Van Swinden Laboratorium [VSL], Delft, The Netherlands,
16. Iowa State University, Ames, Iowa, USA, and
17. Bureau International des Poids et Mesures [BIPM], Sèvres, France.

The CCT met September 12–14, 1989 at the Bureau International des Poids et Mesures (BIPM) in its 17th Session [1] and completed the final details of the new temperature scale, the International Temperature Scale of 1990 (ITS-90) [2]. The CCT then recommended to the CIPM at its meeting on September 26–28, 1989 at the BIPM that the ITS-90 be adopted and made the official scale. The CIPM did adopt the new temperature scale at their meeting [3] and the ITS-90 became the official international temperature scale on January 1, 1990, the same date on which changes affecting certain electrical reference standards were implemented [4]. The ITS-90 supersedes the International Practical Temperature Scale of 1968, Amended Edition of 1975 [IPTS-68(75)] [5] and the 1976 Provisional 0.5 K to 30 K Temperature Scale (EPT-76) [6].

The CCT undertook the development of the ITS-90 because of the deficiencies and limitations of the IPTS-68(75) and completed the scale in accordance with Resolution 7 of the 18th Conférénce Générale des Poid et Mesures [7], which met in October 1987. The deficiencies and limitations of the IPTS-68(75) included its lower limit of 13.81 K, its inaccuracy relative to thermodynamic temperatures, and its non-uniqueness and irreproducibility, especially in the temperature region from *T*_68_ = 903.89 K (*t*_68_ = 630.74 °C) to *T*_68_ = 1337.58 K (*t*_68_ = 1064.43 °C), the region in which the Pt-10%Rh/Pt thermocouple was the standard interpolating instrument.

The ITS-90 extends upward from 0.65 K and temperatures on this scale are in much better agreement with thermodynamic values than are those on the IPTS-68(75) and the EPT-76. The new scale has subranges and alternative definitions in certain ranges that greatly facilitate its use. Furthermore, its continuity, precision, and reproducibility throughout its range are much improved over the corresponding characteristics of the previous scales. The replacement of the thermocouple with the platinum resistance thermometer at temperatures in the range from 630.74 to 961.93 °C of the IPTS-68(75) resulted in the biggest improvement in reproducibility. Also, improvements in radiometric techniques have allowed using the silver freezing point as the reference point for radiation thermometry. This is a lower temperature reference point than was used in the IPTS-68(75).

**Figure 1:**
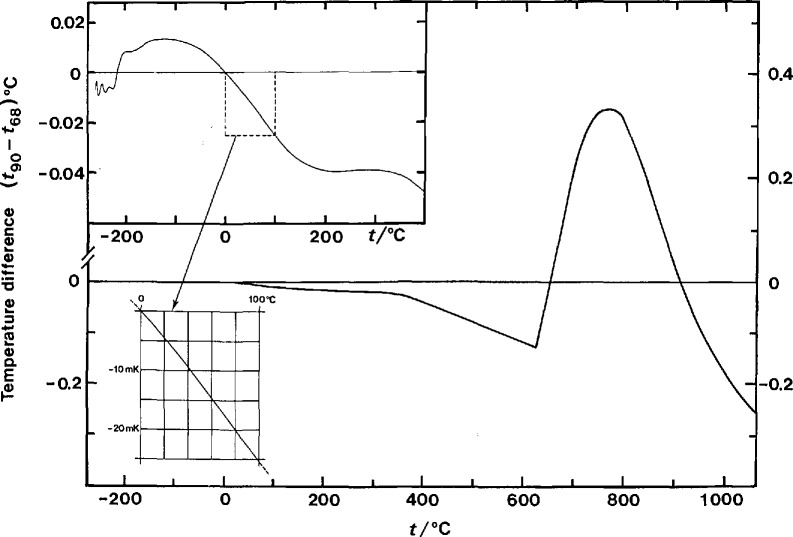
Differences between *t*_90_ and *t*_68_ as a function of *t*_90_, (expressed as *t*).

The change in the temperature scale affects not only technical interests involved directly in thermometry but also those involved with other reference standards, such as electrical standards, if those standards are sensitive to temperature. As examples, standard resistors and standard cells are sensitive to temperature and generally are maintained in constant-temperature environments, at least in national standards laboratories. At the present time, the temperatures of those environments are normally determined with thermometers that have been calibrated on the IPTS-68(75). A given thermodynamic temperature expressed on the ITS-90, however, has a value that is different from that expressed on the IPTS-68(75), as indicated [2,8] in figure 1. A table of differences between temperatures on the ITS-90, *T*_90_ or *t*_90_, and those on the IPTS-68(75), *T*_68_ or *t*_68_, and those on the EPT-76, *T*_76_, is given in the text of the ITS-90. Since temperature values expressed on these scales are different, if the temperature of the environment of a reference standard is adjusted so that *its value when expressed on the ITS-90* has the *same value* as had been used on the IPTS-68(75), there will have been a *change* of the thermodynamic temperature and the value of the reference standard will usually change. Of course, one may not want to change the thermodynamic temperature of the reference standard. In that case, the thermodynamic temperature, as expressed on the IPTS-68(75), can simply be expressed on the ITS-90 (a numerical value different from that on the IPTS-68(75)) and the reference standards will be unaffected. For more details on the effects of the change of the temperature scale on electrical standards, see National Institute of Standards and Technology (NIST) Technical Note 1263 [4].

In addition to the effect on reference standards for measurements, all temperature-sensitive properties that are presently expressed on the IPTS-68(75) may be affected and may require changes in values.

For details on realizations and approximations of the ITS-90, see NIST Technical Note 1265 [9].

## 2. CCT’s 17th Session Principal Decisions

### 2.1 Definition of the ITS-90

The ITS-90 was designed by the CCT in such a manner that temperature values obtained on it do not deviate from the Kelvin thermodynamic temperature values by more than the uncertainties of the latter values at the time the ITS-90 was adopted. Thermodynamic temperature is indicated by the symbol *T* and has the unit known as the kelvin, symbol K. The size of the kelvin is defined to be 1/273.16 of the thermodynamic temperature of the triple point of water.

Temperatures on the ITS-90 can be expressed in terms of the International Kelvin Temperatures, with the symbol *T*_90_, or in terms of the International Celsius Temperatures, with the symbol *t*_90_. The unit of the temperature *T*_90_ is the kelvin, symbol K, and the unit of the temperature *t*_90_ is the degree Celsius, symbol °C. The relation between *T*_90_ and *t*_90_ is:

$$t_{90}/{}^{\circ}C=T_{90}/K-273.15.$$ (1)

The ITS-90 has alternative definitions of *T*_90_ in certain temperature ranges and they have equal status. In measurements of the highest precision made at the same temperature, the alternative definitions may yield detectably different temperature values. Also, at any given temperature between defining fixed points, different interpolating thermometers that meet the specifications of the ITS-90 may indicate different temperature values. The magnitude of the differences resulting from these two sources, however, is sufficiently small to be negligible for all practical purposes.

Temperatures on the ITS-90 are defined in terms of equilibrium states of pure substances (defining fixed points), interpolating instruments, and equations that relate the measured property to *T*_90_. The defining equilibrium states of the pure substances and the assigned temperatures are listed in table 1.

#### 2.1.1 Temperature Range From 0.65 to 5.0 K

Between 0.65 and 3.2 K, the ITS-90 is defined by the vapor pressure-temperature relation of ^3^He, and between 1.25 and 2.1768 K (the *X* point) and between 2.1768 and 5.0 K by the vapor pressure-temperature relations of ^4^He. *T*_90_ is defined by the vapor pressure equations of the form:

$$T_{90}/K=A_{0}+\sum_{i=1}^{9}A_{i}{\left[\left(\ln\left(p/\text{Pa}\right)-B\right)/C\right]}^{i},$$ (2)

with the values of the coefficients *A_i_*, and of the constants *A*_0_, *B*, and *C* of the equations being specified, and given in table 2.

#### 2.1.2 Temperature Range From 3.0 to 24.5561 K

Between 3.0 and 24.5561 K, the ITS-90 is defined in terms of the ^3^He or ^4^He constant volume gas thermometer (CVGT). The thermometer is calibrated at three temperatures—at the triple point of neon (24.5561 K), at the triple point of equilibrium hydrogen (see footnote a in table 1) (13.8033 K), and at a temperature between 3.0 and 5.0 K, the value of which is determined by using either ^3^He or ^4^He vapor pressure thermometry.

For a ^4^He CVGT used between 4.2 K and the triple point of neon (24.5561 K), *T*_90_ is defined by the equation:

$$T_{90}=a+bp+cp^{2},$$ (3)

where *p* is the CVGT pressure and *a*, *b*, and *c* are coefficients that are determined by calibration at the three specified temperatures, but with the additional requirement that the calibration with the vapor pressure thermometer be made at a temperature between 4.2 and 5.0 K.

For a ^4^He CVGT used between 3.0 and 4.2 K, and for a ^3^He CVGT used from 3.0 to 24.5561 K, the non-ideality of the gas must be taken into account, using the appropriate second virial coefficient, *B*_4_(*T*_90_) or *B*_3_(*T*_90_). *T*_90_ is defined in this range by the equation:

$$T_{90}=\frac{a+bp+cp^{2}}{1+B_{x}\left(T_{90}\right)N/V},$$ (4)

where *p* is the CVGT pressure; *a*, *b*, and *c* are coefficients that are determined from calibration at the three defining temperatures; *B_x_*(*T*_90_) refers to *B*_3_(*T*_90_) or *B*_4_(*T*_90_); and *N/V* is the gas density in moles per cubic meter in the CVGT bulb. The values of the second virial coefficients at any given temperature are to be calculated according to equations specified in the official document of the ITS-90 (and also in the NIST Technical Note 1265 [9]).

#### 2.1.3 Temperature Range From 13.8033 to 1234.93 K

Between 13.8033 K (−259.3467 °C) and 1234.93 K (961.78 °C), the ITS-90 is defined in terms of the specified fixed points given in table 1, by resistance ratios of platinum resistance thermometers (PRTs) obtained by calibration at specified sets of the fixed points, and by reference functions and deviation functions of resistance ratios which relate to *T*_90_ between the fixed points.

Temperatures on the ITS-90 are expressed in terms of the ratio *W*(*T*_90_) of the resistance *R*(*T*_90_) at temperature *T*_90_ and the resistance *R*(273.16 K) at the triple point of water, i.e.,

$$W\left(T_{90}\right)=R\left(T_{90}\right)/R\left(273.16\;K\right).$$ (5)

For a PRT to be an acceptable instrument of the ITS-90, its coil must be made from pure platinum and be strain-free. Additionally, the finished PRT must meet one of the following criteria:

W(302.9146K)≥1.11807 (6)

W(234.3156K)≤0.844235. (7)

An acceptable PRT that is to be used to the freezing point of silver must meet the following requirement also:

W(1234.93K)≥4.2844. (8)

The temperature *T*_90_ is calculated from the resistance ratio relation:

$$W\left(T_{90}\right)-W_{r}\left(T_{90}\right)=\Delta W\left(T_{90}\right),$$ (9)

where *W*(*T*_90_) is the observed value, *W*(*T*_90_) is the value calculated from the reference function, and ∆*W*(*T*_90_) is the deviation of the observed *W*(*T*_90_) value of the particular PRT from the reference function value at *T*_90_.

There are two reference functions *W*_r_(*T*_90_), one for the range 13.8033 to 273.16 K and the second for the range 273.15 to 1234.93 K. The deviation ∆*W*(*T*_90_) is obtained as a function of *T*_90_ for various ranges by calibration at specified fixed points. The form of the deviation function depends upon the temperature range of calibration.

#### 2.1.4 Temperature Subrange From 13.8033 to 273.16 K

In the range 13.8033 to 273.16 K, the equation for the reference function *W*_r_(*T*_90_) as a function of *T*_90_ is given by:

$$\begin{array}{l} \ln\left[W_{r}\left(T_{90}\right)\right]=A_{0}+\sum_{i=1}^{12}A_{i}\{[\ln\left(T_{90}/273.16\;K\right)\\ +1.5]/{1.5\}}^{i}. \end{array}$$ (10)

The specified inverse of this equation, equivalent to within ±0.000 1 K, is:

$$T_{90}/273.16\;K=B_{0}+\sum_{i=1}^{15}B_{i}{\left(\frac{{\left[W_{r}\left(T_{90}\right)\right]}^{1/6}-0.65}{0.35}\right)}^{i}.$$ (11)

The values of the constants *A*_0_ and *B*_0_ and values of the coefficients *A_i_* and *B_i_*, of the two equations are listed in table 3.

If the PRT is to be used throughout the range from 13.8033 to 273.16 K, it must be calibrated at the triple points of equilibrium hydrogen (13.8033 K), neon (24.5561 K), oxygen (54.3584 K), argon (83.8058 K), mercury (234.3156 K), and water (273.16 K) and at two additional temperatures very close to 17.0 and 20.3 K. The temperatures of calibration at 17.0 and 20.3 K may be determined by using either a CVGT or the vapor pressure-temperature relation of equilibrium hydrogen. When the CVGT is used, the two temperatures must be within the ranges 16.9 to 17.1 K and 20.2 to 20.4 K, respectively. When the equilibrium hydrogen vapor pressure thermometer is used, the two temperatures must be within the ranges 17.025 to 17.045 K and 20.26 to 20.28 K, respectively. The temperatures of the equilibrium hydrogen vapor pressure thermometer are determined from the values of the hydrogen vapor pressure, *p*, and the equations:

$$T_{90}/K-17.035=\left(p/\text{kPa}-33.3213\right)/13.32$$ (12)

$$T_{90}/K-20.27=\left(p/\text{kPa}-101.292\right)/30.$$ (13)

Depending upon the temperature range of application, PRTs may be calibrated from 273.16 K down to 13.8033 K (triple point of equilibrium hydrogen), down to 24.5561 K (triple point of neon), down to 54.3584 K (triple point of oxygen), or down to 83.8058 K (triple point of argon).

The deviation function for calibration in the range 13.8033 to 273.16 K is given by the relation:

$$\begin{array}{c} \Delta W_{1}\left(T_{90}\right)=W\left(T_{90}\right)-W_{r}\left(T_{90}\right)=a_{1}\left[W\left(T_{90}\right)-1\right]\\ +b_{1}{\left[W\left(T_{90}\right)-1\right]}^{2}+\sum_{i=1}^{5}c_{i}{\left[\ln W\left(T_{90}\right)\right]}^{i+n}, \end{array}$$ (14)

with *n* = 2. The coefficients *a*_1_, *b*_1_, and the five *c_i_*’s of the deviation function are obtained by calibration at all of the above eight temperatures, including that at the triple point of water. The values of *W*_r_(*T*_90_) are obtained from the reference function for this range. Although the official text of the ITS-90 does not assign subscripts to the coefficients *a* and *b*, nor does it designate the deviation equations by the symbols ∆*W*_m_(*T*_90_), where in eq (14)*m* = 1, these designations will be used in this paper for clarity and for ease of reference. In any case, some such terminology must be used in PRT calibration reports and this was chosen for convenience.

#### 2.1.5 Subrange From 24.5561 to 273.16 K

The deviation function for calibration in this range is given by the relation:

$$\begin{array}{c} \Delta W_{2}\left(T_{90}\right)=W\left(T_{90}\right)-W_{r}\left(T_{90}\right)=a_{2}\left[W\left(T_{90}\right)-1\right]\\ +b_{2}{\left[W\left(T_{90}\right)-1\right]}^{2}+\sum_{i=1}^{3}c_{i}{\left[\ln W\left(T_{90}\right)\right]}^{i+n}, \end{array}$$ (15)

where the exponent *n* has the value *n* =0. The coefficients *a*_2_, *b*_2_, and *c_i_*, of this deviation function are obtained by calibrating the PRT at the triple points of equilibrium hydrogen (13.8033 K), neon (24.5561 K), oxygen (54.3584 K), argon (83.8058 K), mercury (234.3156 K) and water (273.16 K). The values of *W*_r_(*T*_90_) are obtained from the reference function.

#### 2.1.6 Subrange From 54.3584 to 273.16 K

The deviation function for calibration in this range is given by the relation:

$$\begin{array}{l} \Delta W_{3}\left(T_{90}\right)=a_{3}\left[W\left(T_{90}\right)-1\right]+b_{3}{\left[W\left(T_{90}\right)-1\right]}^{2}\\ \;+c_{1}{\left[\ln W\left(T_{90}\right)\right]}^{1+n}, \end{array}$$ (16)

where the exponent *n* has the value *n* = 1. The coefficients *a*_3_, *b*_3_, and *c*_1_ of this deviation function are obtained by calibrating the PRT at the triple points of oxygen (54.3584 K), argon (83.8058 K), mercury (234.3156 K), and water (273.16 K). The values of *W*_r_(*T*_90_) are obtained from the reference function.

#### 2.1.7 Subrange From 83.8058 to 273.16 K

The deviation function for calibration in this range is given by the relation:

$$\begin{array}{l} \Delta W_{4}\left(T_{90}\right)=a_{4}\left[W\left(T_{90}\right)-1\right]\\ \;+b_{4}\left[W\left(T_{90}\right)-1\right]\ln W\left(T_{90}\right). \end{array}$$ (17)

The coefficients *a*_4_ and *b*_4_ of this deviation function are obtained by calibrating the PRT at the triple points of argon (83.8058 K), mercury (234.3156 K), and water (273.16 K). The values of *W*_r_(*T*_90_) are obtained from the reference function.

#### 2.1.8 Temperature Subrange From 273.15 to 1234.93 K

In the range 273.15 to 1234.93 K, the equation for the reference function *W*_r_(*T*_90_) is given by:

$$W_{r}\left(T_{90}\right)=C_{0}+\sum_{i=1}^{9}C_{i}{\left(\frac{T_{90}/K-754.15}{481}\right)}^{i}.$$ (18)

The specified inverse of this equation, equivalent to within ±0.000 13 K, is:

$$T_{90}/K-273.15=D_{0}+\sum_{i=1}^{9}D_{i}{\left(\frac{W_{r}\left(T_{90}\right)-2.64}{1.64}\right)}^{i}.$$ (19)

The values of the constants *C*_0_ and *D*_0_ and of the coefficients *C_i_* and *D_i_* for these equations are listed in table 3.

If the PRT is to be used over this entire subrange (273.15 to 1234.93 K), it must be calibrated at the triple point of water (273.16 K) and at the freezing points of tin (505.078 K), zinc (692.677 K), aluminum (933.473 K), and silver (1234.93 K).

The deviation function is given by the relation:

$$\begin{array}{l} \Delta W_{6}\left(T_{90}\right)=W\left(T_{90}\right)-W_{r}\left(T_{90}\right)=a_{6}\left[W\left(T_{90}\right)-1\right]\\ \;+b_{6}{\left[W\left(T_{90}\right)-1\right]}^{2}+c_{6}{\left[W\left(T_{90}\right)-1\right]}^{3}\\ \;+d{\left[W\left(T_{90}\right)-W\left(933.473\;K\right)\right]}^{2}. \end{array}$$ (20)

The values of the coefficients *a*_6_, *b*_6_, and *c*_6_ are determined from the measured deviations ∆*W*_r_(*T*_90_) of *W*(*T*_90_) from the reference values *W*_r_(*T*_90_) at the freezing points of tin (505.078 K), zinc (692.677 K) and aluminum (933.473 K). The coefficient *d* is determined from these values of the coefficients *a*_6_, *b*_6_, and *c*_6_ and the deviation ∆*W*(*T*_90_) of *W*(*T*_90_) from the reference value *W*_r_(*T*_90_) at the freezing point of silver. The coefficient *d* in this equation is used only for those temperature measurements in the range from the freezing point of aluminum to the freezing point of silver. For temperature measurements below the freezing point of aluminum, *d* = 0.

PRTs may be calibrated for use over the whole range (273.15 to 1234.93 K) or for shorter ranges by calibrations at fixed points between 273.15 K and the upper limit of 933.473 K (freezing point of aluminum, 660.323 °C), of 692.677 K (freezing point of zinc, 419.527 °C), of 505.078 K (freezing point of tin, 231.928 °C), of 429.7485 K (freezing point of indium, 156.5985 °C), or of 302.9146 K (melting point of gallium, 29.7646 °C).

#### 2.1.9 Subrange From 273.15 to 933.473 K

For application in this range, the PRT is calibrated at the triple point of water (273.16 K), and at the freezing points of tin (505.078 K), zinc (692.677 K), and aluminum (933.473 K). The deviation function is given by the relation:

$$\begin{array}{l} \Delta W_{7}\left(T_{90}\right)=a_{7}\left[W\left(T_{90}\right)-1\right]+b_{7}{\left[W\left(T_{90}\right)-1\right]}^{2}\\ \;+c_{2}{\left[W\left(T_{90}\right)-1\right]}^{3}. \end{array}$$ (21)

The coefficients *a*_7_, *b*_7_, and *c*_7_, identical to *a*_6_, *b*_6_, and *c*_6_, respectively, are determined from the deviations ∆*W*(*T*_90_) of *W*(*T*_90_) from the reference values ∆*W*_r_(*T*_90_) at the freezing points of tin (505.078 K), zinc (692.677 K), and aluminum (933.473 K).

#### 2.1.10 Subrange From 273.15 to 692.677 K

For application in this range, the PRT is calibrated at the triple point of water (273.16 K), and at the freezing points of tin (505.078 K) and zinc (692.677 K). The deviation function is given by the relation:

$$\Delta W_{8}\left(T_{90}\right)=a_{8}\left[W\left(T_{90}\right)-1\right]+b_{8}{\left[W\left(T_{90}\right)-1\right]}^{2}.$$ (22)

The coefficients *a*_8_ and *b*_8_ are determined from the deviations ∆*W*(*T*_90_) of *W*(*T*_90_) from the reference values *W*(*T*_90_) at the freezing points of tin (505.078 K) and zinc (692.677 K).

#### 2.1.11 Subrange From 273.15 to 505.078 K

For application in this range, the PRT is calibrated at the triple point of water (273.16 K), and at the freezing points of indium (429.7485 K) and tin (505.078 K). The form of the deviation function is the same as that for the subrange 273.15 to 692.677 K, i.e.,

$$\Delta W_{8}\left(T_{90}\right)=a_{9}\left[W\left(T_{90}\right)-1\right]+b_{9}{\left[W\left(T_{90}\right)-1\right]}^{2}.$$ (23)

The coefficients *a*_9_ and *b*_9_ are determined from the deviations ∆*W*(*T*_90_) of *W*(*T*_90_) from the reference values *W*_r_(*T*_90_) at the freezing points of indium (429.7485 K) and tin (505.078 K).

#### 2.1.12 Subrange From 273.15 to 429.7485 K

For application in this range, the PRT is calibrated at the triple point of water (273.16 K) and at the freezing point of indium (429.7485 K). The deviation function is:

$$\Delta W_{10}\left(T_{90}\right)=a_{10}\left[W\left(T_{90}\right)-1\right].$$ (24)

The coefficient *a*_10_ is determined from the deviation ∆*W*(*T*_90_) of *W*(*T*_90_) from the reference value *W*_r_(*T*_90_) at the freezing point of indium (429.7485 K).

#### 2.1.13 Subrange From 273.15 to 302.9146 K

For application in this range, the PRT is calibrated at the triple point of water (273.16 K) and at the melting point of gallium (302.9146 K). The deviation function is:

$$\Delta W_{11}\left(T_{90}\right)=a_{11}\left[W\left(T_{90}\right)-1\right].$$ (25)

The coefficient *a*_11_ is determined from the deviation ∆*W*(*T*_90_) of *W*(*T*_90_) from the reference value *W*_r_(*T*_90_) at the melting point of gallium (302.9146 K).

#### 2.1.14 Subrange From 234.3156 to 302.9146 K

For application in this range, the PRT is calibrated at the triple points of mercury (234.3156 K) and water (273.16 K), and at the melting point of gallium (302.9146 K). The form of the deviation function is the same as that for the subrange 273.15 to 692.677 K, i.e.,

$$\Delta W_{5}\left(T_{90}\right)=a_{5}\left[W\left(T_{90}\right)-1\right]+b_{5}{\left[W\left(T_{90}\right)-1\right]}^{2}.$$ (26)

The coefficients *a*_5_ and *b*_5_ are determined from the deviations ∆*W*(*T*_90_) of *W*(*T*_90_) from the reference values *W*_r_(*T*_90_) at the triple point of mercury (234.3156 K) and at the melting point of gallium (302.9146 K). The reference values *W*(*T*_90_) must be calculated from the relevant reference function, both reference functions being required to cover this range.

#### 2.1.15 Temperature Range Above 1234.93 K

At temperatures above 1234.93 K, T_90_ is defined by the relation:

$$\frac{L_{\lambda }\left(T_{90}\right)}{L_{\lambda }\left[T_{90}\left(X\right)\right]}=\frac{\exp\left[c_{2}/\lambda T_{90}\left(X\right)\right]-1}{\exp\left[c_{2}/\lambda T_{90}\right]-1},$$ (27)

in which *L*_λ_(*T*_90_) and *L*_λ_[*T*_90_(X)] are the spectral concentrations of the radiance of a blackbody at wavelength λ (in vacuum) at *T*_90_ and at *T*_90_(X), respectively. *T*_90_(X) refers to either the silver freezing point [*T*_90_(Ag) = 1234.93 K], the gold freezing point [*T*_90_(Au) = 1337.33 K] or the copper freezing point [*T*_90_(Cu) = 1357.77 K]. *c*_2_ = 0.014388 m·K. Although the freezing-point temperature of silver is the junction point of platinum resistance thermometry and radiation thermometry, it is believed that the *T*_90_ values of the freezing points of silver, gold and copper are sufficiently self-consistent that the use of any one of them as the reference temperature *T*_90_(X) will not result in any significant difference in the measured values of *T*_90_ from what would be obtained if only the silver freezing point were used.

### 2.2 Recommendations of the CCT

Three recommendations were adopted by the CCT at its 17th Session. These recommendations were considered by the CIPM and Recommendation T1 (1989) of the CCT was adopted as Recommendation 5 (CI-89) of the CIPM. Recommendations T2 (1989) and T3 (1989) of the CCT were noted by the CIPM as CCT recommendations. The CCT recommendations were as follows:

#### Recommendation T1 (1989) The International Temperature Scale of 1990

The Comité Consultatif de Thermométrie (CCT) acting in accordance with Resolution 7 of the 18^e^ CGPM has generated the International Temperature Scale of 1990 (ITS-90) in order to supersede the International Practical Temperature Scale of 1968 (IPTS-68).

The CCT notes that, by comparison with the IPTS-68, the ITS-90

—extends to lower temperatures, down to 0.65 K, and hence also supersedes the EPT-76,
—is in substantially better agreement with corresponding thermodynamic temperatures,
—has much improved continuity, precision, and reproducibility throughout its range and
—has subranges and alternative definitions in certain ranges which greatly facilitate its use.

The CCT also notes that, to accompany the text of the ITS-90 there will be two further documents, the Supplementary Information for the ITS-90 and Techniques for Approximating the ITS-90. These documents will be published by the BIPM and periodically updated.

The CCT recommends

—that on January 1, 1990 the ITS-90 come into force and
—that from this same date the IPTS-68 and the EPT-76 be abrogated.

#### Recommendation T2 (1989) Reference Tables for Thermocouples and Industrial Platinum Resistance Thermometers

The Comité Consultatif de Thermométrie,

considering
  —that the introduction of the International Temperature Scale of 1990 (ITS-90) will lead to an urgent requirement for new reference tables for both thermocouples and industrial platinum resistance thermometers,
*requests* its Working Group 2
  —to collaborate with national laboratories in the rapid preparation of new reference tables taking into account not only the change from IPTS-68 to ITS-90 but also new information on the behavior of thermocouples and industrial platinum resistance thermometers,
recommends
  —that these new tables be used as the basis for new national and international reference tables for thermocouples and industrial platinum resistance thermometers and
—that meanwhile the existing reference tables based upon IPTS-68 should be used in conjunction with the table of differences *T*_90_ − *T*_68_ which appears in the ITS-90.

(Note: the table of differences *T*_90_ − *T*_68_ referred to here may be obtained also from NIST from the author of this article).

#### Recommendation T3 (1989) The Uncertainty Inherent in the Realization of the International Temperature Scale of 1990

The Comité Consultatif de Thermométrie, *considering* the requirement for assigning an uncertainty to the numerical value of any temperature on the International Temperature Scale of 1990 (ITS-90),

*encourages* national laboratories to

- quantify the uncertainties in the fixed point realizations,
- quantify the uncertainties resulting from the use of the specified interpolating instruments of ITS-90,
- develop the mathematical procedures describing the propagation of these uncertainties to any intermediate temperature.

## 3. Conclusion

Although the uncertainties in the values of thermodynamic temperatures above 100 °C used in the definition of the ITS-90 were larger than desired and larger than had been anticipated a few years ago, the agreement of temperatures on the ITS-90 with thermodynamic temperatures is nevertheless a significant improvement over that of previous scales. The replacement of the thermocouple with the platinum resistance thermometer as the standard instrument of the scale at temperatures in the IPTS-68(75) range from 630.74 to 961.93 °C has improved the reproducibility over that of the IPTS-68(75) significantly. Also, advances in radiometric techniques have improved the precision of measurements in radiation thermometry. The precision of the scale, or what has been called the non-uniqueness of the scale, is significantly improved over that of the IPTS-68(75), as is also the scale’s continuity. The extension of the scale downward in temperature to 0.65 K and the use of subranges over which thermometers may be calibrated make the ITS-90 more useful and much more flexible than were the previous scales.

## Figures and Tables

**Table 1:** Defining fixed points of the ITS-90

Material^a^	Equilibrium state^b^	Temperature	
		*T*_90_(K)	*t*_90_(°C)
^3^He and ^4^He	VP	3 to 5	−270.15 to
			−268.15
e-H_2_	TP	13.8033	−259.3467
e-H_2_ (or He)	VP (or CVGT)	≈17	≈ −256.15
e-H_2_ (or He)	VP (or CVGT)	≈20.3	≈ −252.85
Ne^c^	TP	24.5561	−248.5939
O_2_	TP	54.3584	−218.7916
Ar	TP	83.8058	−189.3442
Hg^c^	TP	234.3156	−38.8344
H_2_O	TP	273.16	0.01
Ga^c^	MP	302.9146	29.7646
In^c^	FP	429.7485	156.5985
Sn	FP	505.078	231.928
Zn	FP	692.677	419.527
Al^c^	FP	933.473	660.323
Ag	FP	1234.93	961.78
Au	FP	1337.33	1064.18
Cu^c^	FP	1357.77	1084.62

a e-H_2_ indicates equilibrium hydrogen, that is, hydrogen with the equilibrium distribution of its ortho and para states. Normal hydrogen at room temperture contains 25% para hydrogen and 75% ortho hydrogen.

b VP indicates vapor pressure point; CVGT indicates constant volume gas thermometer point; TP indicates triple point (equilibrium temperature at which the solid, liquid and vapor phases coexist); FP indicates freezing point and MP indicates melting point (the FP and the MP are equilibrium temperatures at which the solid and liquid phases coexist under a pressure of 101 325 Pa, one standard atmosphere). The isotopic composition is that naturally occurring.

c Previously, these were secondary fixed points.

**Table 2:** Values of the coefficients *A_i_*, and of the constants *A*_0_, *B*, and *C* for the ^3^He and ^4^He vapor pressure equations and the temperature range for which each equation is valid

Coef. or constant	^3^He 0.65 to 3.2 K	^4^He 1.25 to 2.1768 K	^4^He 2.1768 to 5.0 K
*A*_0_	1.053 447	1.392 408	3.146 631
*A*_1_	0.980 106	0.527 153	1.357 655
*A*_2_	0.676 380	0.166 756	0.413 923
*A*_3_	0.372 692	0.050 988	0.091 159
*A*_4_	0.151656	0.026 514	0.016 349
*A*_5_	−0.002 263	0.001 975	0.001 826
*A*_6_	0.006 596	−0.017 976	−0.004 325
*A*_7_	0.088 966	0.005 409	−0.004 973
*A*_8_	−0.004 770	0.013 259	0
*A*_9_	−0.054 943	0	0
*B*	7.3	5.6	10.3
*C*	4.3	2.9	1.9

**Table 3:** Values of the coefficients *A_i_*, *B_i_*, *C_i_*, and *D_i_* and of the constants *A*_0_, *B*_0_, *C*_0_, and *D*_0_, in the reference functions, eqs (10) and (18), and in the inverse functions approximating them, given by eqs (11) and (19)

Constant or coefficient	Value	Constant or coefficient	Value
*A*_0_	−2.135 347 29	*B*_0_	0.183 324 722
*A*_1_	3.183 247 20	*B*_1_	0.240 975 303
*A*_2_	−1.801 435 97	*B*_2_	0.209 108 771
*A*_3_	0.717 272 04	*B*_3_	0.190 439 972
*A*_4_	0.503 440 27	*B*_4_	0.142 648 498
*A*_5_	−0.618 993 95	*B*_5_	0.077 993 465
*A*_6_	−0.053 323 22	*B*_6_	0.012 475 611
*A*_7_	0.280 213 62	*B*_7_	−0.032 267 127
*A*_8_	0.107 152 24	*B*_8_	−0.075 291 522
*A*_9_	−0.293 028 65	*B*_9_	−0.056 470 670
*A*_10_	0.044 598 72	*B*_10_	0.076 201 285
*A*_11_	0.118 686 32	*B*_11_	0.123 893 204
*A*_12_	−0.052 481 34	*B*_12_	−0.029 201 193
		*B*_13_	−0.091 173 542
		*B*_14_	0.001 317 696
		*B*_15_	0.026 025 526
*C*_0_	2.781 572 54	*D*_0_	439.932 854
*C*_1_	1.646 509 16	*D*_1_	472.418 020
C_2_	−0.137 143 90	*D*_2_	37.684 494
*C*_3_	−0.006 497 67	*D*_3_	7.472 018
*C*_4_	−0.002 344 44	*D*_4_	2.920 828
*C*_5_	0.005 118 68	*D*_5_	0.005 184
*C*_6_	0.001 879 82	*D*_6_	−0.963 864
*C*_7_	−0.002 044 72	*D*_7_	−0.188 732
*C*_8_	−0.000 461 22	*D*_8_	0.191 203
*C*_9_	0.000 457 24	*D*_9_	0.049 025

## References

[1] BIPM Com (1989). Cons Thermométrie.

[2] (1990). The International Temperature Scale of 1990. Metrologia.

[3] octobre (1989). Procès-Verbaux des séances du Comité International des Poids et Mesures.

[4] Belecki NB, Dziuba RF, Field BF, Taylor BN (1989). Guidelines for Implementing the New Representations of the Volt and Ohm Effective January 1, 1990.

[5] (1976). The International Temperature Scale of 1968, Amended Edition of 1975. Metrologia.

[6] (1979). The 1976 Provisional 0.5 K to 30 K Temperature Scale. Metrologia.

[7] (1987).

[8] Quinn TJ (1989). Metrologia.

[9] Mangum BW, Furukawa GT (1990). Guidelines for Realizing the International Temperature Scale of 1990.

